# Ceruloplasmin and the Extent of Heart Failure in Ischemic and Nonischemic Cardiomyopathy Patients

**DOI:** 10.1155/2013/348145

**Published:** 2013-05-28

**Authors:** Yifei Xu, Haiyan Lin, Ying Zhou, Gang Cheng, Geng Xu

**Affiliations:** Department of Cardiology, Second Affiliated Hospital, Zhejiang University, College of Medicine, No. 88 Jiefang Road, Hangzhou, Zhejiang 310009, China

## Abstract

*Objective*. Ceruloplasmin was elevated in patients with coronary heart disease, but the relationship between ceruloplasmin and heart failure was still unknown. We aimed to evaluate ceruloplasmin in heart failure patients and assess association between ceruloplasmin and the extent of heart failure. *Methods and Results*. 202 heart failure patients were divided into ischemic (78 with coronary stenosis) and nonischemic groups (124 without coronary stenosis). 94 subjects without heart failure were included as controls. The extent of heart failure was defined according to NYHA classification. Ceruloplasmin levels in ischemic (*P* < 0.001) and nonischemic groups (*P* < 0.001) were higher than those in control group. Ceruloplasmin had a positive linear correlation with C-reactive protein (*P* < 0.01) and a negative linear correlation with LVEF (*P* < 0.05). In nonischemic group, CP levels were significantly different among different NYHA subgroups (*P* < 0.05). The correlation between ceruloplasmin and extent of heart failure was calculated by binary logistic regression. Ceruloplasmin showed an independent association with the extent of heart failure in nonischemic cardiomyopathy patients (*P* < 0.05). *Conclusions*. Ceruloplasmin was significantly elevated in patients with ischemic or nonischemic cardiomyopathy and had linear correlation with C-reactive protein and LVEF. In nonischemic cardiomyopathy patients, the ceruloplasmin value was an independent biomarker associated with the extent of heart failure.

## 1. Introduction

During the progression of cardiomyopathy, heart failure was gradually aggravated and became the major cause of unplanned hospitalization. The mortality of heart failure is still high in the present therapy, and brain natriuretic peptide, which is elevated in acute heart failure, could be false positive in pulmonary disease, like pulmonary embolism or chronic obstructive pulmonary disease.

Metal metabolism, metalloenzymes, and their activities of oxidation and inflammation are involved in progression of heart failure [1]. Many studies have shown that ceruloplasmin (CP) levels were elevated in patients with cardiovascular disorders including arteriosclerosis [2], coronary heart disease [3, 4], and myocardial infarction [5]. In the ischemic heart failure, CP is recognized as an inflammation-related marker protein for its predictive value [6]. Elsherif et al. [7] verified that copper deficiency leads to cardiac dysfunction. Correale et al. [8] demonstrated that the CP value is a significant marker of acute heart failure in patients with ST segment elevated myocardial infarction. Serum CP might be an independent risk factor for ischemic cardiac disease [9]. However, several studies still suspected the association [10], and the relationship between CP and heart failure was still unclear in either ischemic or nonischemic cardiomyopathy.

Our study aimed to evaluate the predictive value of serum ceruloplasmin in patients with ischemic and nonischemic cardiomyopathies, and assess the association between CP levels and the extent of heart failure, CP levels and other parameters in both ischemic and nonischemic cardiomyopathy patients.

## 2. Material and Methods

### 2.1. Patient Recruitment

Between December 2009 and April 2011, a total of 202 patients were recruited consecutively from those who were admitted and diagnosed as heart failure patients according to New York Heart Association (NYHA) classification at the Department of Cardiology, Second Affiliated Hospital of Zhejiang University College of Medicine. Patients with advanced renal disease (serum creatinine >2 mg/dL or those on dialysis), noncardiac acute inflammatory states, known malignancies, or liver dysfunction resulting from organic hepatic diseases were excluded. However, patients with diabetes mellitus, hypercholesterolemia, hypertension, and transient liver dysfunction induced by circulatory congestion in congestive heart failure were enrolled in the study. Patients who had coronary stenosis findings in coronary angiography belonged to ischemic cardiomyopathy group, and the other patients belonged to nonischemic cardiomyopathy group. 94 subjects without organic heart disease were included in the control group. Other risk factors were balanced among three groups. The extent of heart failure is defined according to the NYHA functional classification system: NYHA I (normal)—no limitation of physical activity. Ordinary physical activity does not cause undue fatigue, palpitation or dyspnea; NYHA II (mild)—slight limitation of physical activity. Comfortable at rest, but ordinary physical activity results in fatigue, palpitation or dyspnea; NYHA III (moderate)—marked limitation of physical activity. Comfortable at rest, but less than ordinary activity causes fatigue, palpitation, or dyspnea; NHYA IV (severe)—unable to carry out any physical activity without discomfort. Symptoms of heart failure are present at rest. If any physical activity is undertaken, discomfort increases.

All patients enrolled in the study were given informed consent, and the hospital ethics committee approved the study protocol.

### 2.2. Clinical Data Collection

For each patient, the clinical characteristics were collected, including age, gender, height, weight, history of hypertension, diabetes mellitus, cigarette smoking, and NYHA classification. Medications taken (cardiotonic, diuretic, nitrate, angiotensin-converting enzyme inhibitor/angiotensin receptor blocker, *β*-blocker, calcium channel blocker, aspirin/clopidogrel, and statin) were also collected. BMI (body mass index) is defined as the individual's weight (kg) divided by square of height (m).

Echocardiography was performed for each subject and parameters were selected for analysis: LVEF (%)—left-ventricular ejection fraction, LVIDd (cm)—left-ventricular internal diameter at end diastole, and LA (cm)—left atrium.

### 2.3. Blood Sample Analysis

Blood samples were collected on admission, using pyrogen-free tubes containing EDTA for plasma test and sterile tubes containing gel for serum separation (BD, USA) and then immediately centrifuged at 4000 rpm for 10 min at 4°C. All the samples were analyzed in laboratory of Second Affiliated Hospital of Zhejiang University College of Medicine. The results of blood biochemical test including total cholesterol, low-density lipoprotein cholesterol, high-density lipoprotein cholesterol, triglycerides, glucose, creatinine, C-reactive protein, troponin I, creatine kinase-MB, and CP were collected and analyzed.

The levels of serum ceruloplasmin were measured by nephelometry [11] (Beckman Coulter, Immage 800, USA).

### 2.4. Statistical Analysis

Continuous variables were given as mean ± standard deviation. Categorical variables were defined as percentage. Data were tested for normal distribution using the Kolmogorov-Smirnov test. Student's *t*-test and one-way analysis of variance were used for continuous variables and the *χ* ^2^ for the categorical variables. The correlation between ceruloplasmin and other parameters was evaluated by Pearson correlation. The NYHA II and III subgroups were combined as mild group, NYHA IV was the severe group, and then association between the clinical parameters and the extent of heart failure was assessed by binary logistic regression. All tests of significance were two tailed. Statistical significance was defined as *P* value less than 0.05.

## 3. Results

### 3.1. Patient Enrollment and Group Characterization

78 heart failure patients with coronary stenosis and 124 heart failure patients without coronary stenosis were included in ischemic and nonischemic groups, respectively. 94 subjects without heart failure were included as controls. Epidemiological characteristics and biochemical parameters of the patients according to the three groups are described in Table 1. When compared with the control group, both the ischemic and nonischemic cardiomyopathy groups were older, had higher levels of creatinine, uric acid, C-reactive protein, homocysteic acid, LVIDd, and LA, and had lower levels of high-density lipoprotein cholesterol and LVEF. Prevalence of hypertension, diabetes and usage of cardiovascular medication were also higher in both ischemic and nonischemic cardiomyopathy groups than in control group. When compared with ischemic cardiomyopathy group, the nonischemic cardiomyopathy had higher levels of uric acid, LVIDd, LA, higher prevalence of diabetes and smoking, and lower levels of triglycerides and LVEF. The nonischemic group still has higher levels of alanine aminotransferase (ALT) and aspartate aminotransferase (AST) than ischemic and control groups.

To assess the relationship between ceruloplasmin and other parameters, the Pearson linear correlation was evaluated in all the subjects in Table 2. The ceruloplasmin had a significant positive linear correlation with ALT, AST, uric acid, C-reactive protein, LVIDd, and LA (*P* < 0.01). The ceruloplasmin also had a negative linear correlation with LVEF (*P* < 0.05).

### 3.2. Ceruloplasmin and the Extent of Heart Failure

**Figure 1:**
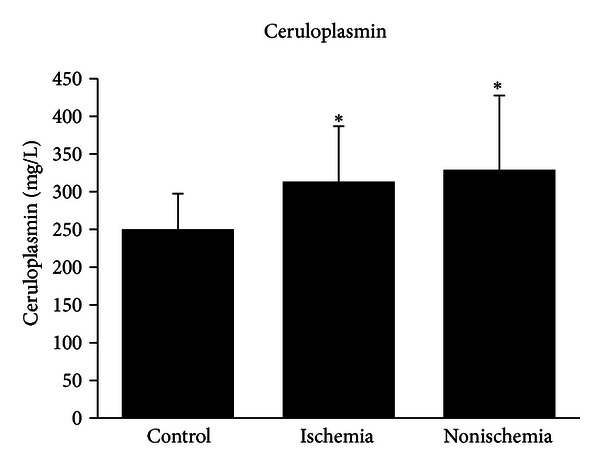
Ceruloplasmin levels in control, ischemic, and nonischemic cardiomyopathy groups. CP levels in ischemic and nonischemic cardiomyopathy groups were higher than those in the control group (*P* < 0.001). No difference was found between the ischemic and nonischemic cardiomyopathy groups. *P < 0.001 versus control group.

**Figure 2:**
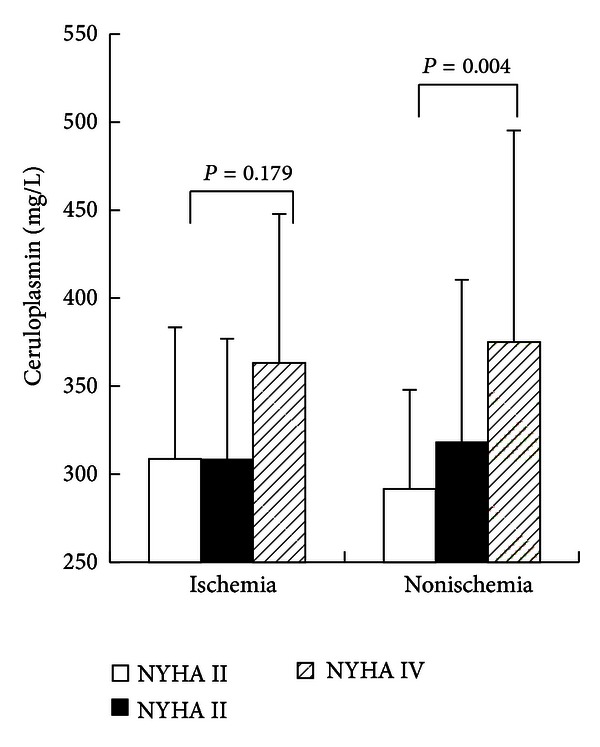
Ceruloplasmin levels in NYHA II, NYHA III, and NYHA IV subgroups. There was no significant difference among different NYHA subgroups in ischemic cardiomyopathy patients (*P* > 0.05). In nonischemic cardiomyopathy patients, the CP levels showed statistical difference among NYHA II, NYHA III, and NYHA IV subgroups (*P* < 0.05).

Mean CP levels were 250.01 ± 47.54, 313.06 ± 73.60, and 328.80 ± 98.91 mg/L, for patients in the control, ischemic cardiomyopathy, and nonischemic cardiomyopathy groups, respectively. CP levels in ischemic (*P* < 0.001) and nonischemic cardiomyopathy groups (*P* < 0.001) were higher than those in the control group (Figure 1), whereas there was no significant difference between the ischemic and nonischemic groups. When CP levels were compared among the different NYHA groups in patients with ischemic cardiomyopathy, there was no significant difference among three groups (308.69 ± 74.89,308.68 ± 68.36, and  363.14 ± 84.65, *P* > 0.05) (Figure 2). In nonischemic cardiomyopathy patients, CP levels were significantly different among NYHA II, NYHA III, and NYHA IV groups (291.81 ± 56.28, 318.54 ± 91.98, and 375.03 ± 120.11, *P* < 0.05) (Figure 2).

### 3.3. Ceruloplasmin and the Extent of Heart Failure in Nonischemic Group

According to results previous mentioned, we tried to identify the relation between ceruloplasmin and the extent of heart failure in nonischemic group. All the related parameters were compared, and the results showed that AST, uric acid, CKMB, CRP, and LVEF were significantly different among NYHA II, NYHA III, and NYHA IV groups (Table 3). Because these parameters could not be balanced among groups, we combined the NYHA II and NYHA III patients as mild group, and then binary logistic regression model was applied between mild group and NHYA IV group. The results showed that only CP levels (*χ* ^2^ = 4.489, odds ratio (OR) = 1.010, 95% confidence interval (CI): 1.001–1.019, *P* = 0.034) and age (*P* = 0.046) were correlated with the extent of heart failure. The correlation between the CP levels and the extent of heart failure was independent of gender, smoking, alcohol taking, hypertension, diabetes mellitus, AST, uric acid, CKMB, CRP, LVEF, and other parameters (Table 4).

## 4. Discussion

Ceruloplasmin (CP) is a 132-kDa plasma glycoprotein which binds 95% of the total circulating copper in healthy adults [12]. The liver is the major source of serum CP in adults [13]. The synthesis of serum CP in hepatic cells is increased by proinflammatory agonists [14–16]. The recent research has indicated that increased ceruloplasmin concentrations were associated with an increased risk of myocardial infarction and stroke [17, 18]. To the best of our knowledge, this is the first study on the relationship between CP levels and the extent of heart failure, which indicated that the CP levels were significantly high in patients with ischemic or nonischemic cardiomyopathy. In nonischemic cardiomyopathy patients, the CP value was an independent biomarker associated with the extent of heart failure.

During the progression of heart failure, the inflammatory and oxidative reactions were activated and enhanced as a protective mechanism. So we hypothesized that CP, which was an index of the metal metabolism, still could be the inflammatory and oxidative biomarker in heart failure. The results proved our hypothesis. The CP level was elevated in patients with cardiomyopathy and showed the positive linear correlation with uric acid and C-reactive protein and negative linear correlation with left-ventricular ejection fraction, which meant that CP was increased with other acute-phase biomarkers when heart function got worse. Then we compared the CP levels of different NYHA levels in ischemic and nonischemic cardiomyopathy groups, respectively.

In ischemic heart disease, ischemic injury to cardiac muscle induced the inflammation and redox toxic reactions and the injury ran through every stage of ischemic cardiomyopathy. Copper might be involved in the redox toxic reaction [19]. CP is elevated by proinflammatory agonists to play a role of cytoprotection [20] and should be elevated when heart function was aggravated in our hypothesis. However, when we compared the CP value among different NYHA levels in ischemic cardiomyopathy group, no statistical difference was found. We believed that it was related to the small number of patients included, because only 7 patients were included in NYHA IV subgroup and the mean CP value in NYHA IV patients was much higher than that in other subgroups.

In nonischemic cardiomyopathy patients, heart failure is always accompanied with tissue hypoxia. The hypoxia was exacerbated with low-pulse oxygen saturation clinically when heart failure was aggravated. CP synthesis was increased as a defense mechanism in response to hypoxia [21]. Our results showed the statistical difference among different NYHA levels in nonischemic group, suggesting that CP's ferroxidase activity and metal metabolism might play important roles. However, when we analyzed other risk factors and parameters related to the extent of heart failure, AST, uric acid, CKMB, CRP, and LVEF were not well balanced among subgroups, and so the logistic regression was given. The results showed that CP value was well correlated with the extent of heart failure, and this correlation was independent of AST, uric acid, CKMB, LVEF, and even C-reactive protein which is a sensitive marker of inflammation. The correlation was also independent of gender, hypertension, diabetes mellitus, and other risk factors. Age was the only risk factor related to the extent of heart failure.

## 5. Conclusions

We concluded that CP levels were significantly high in patients with ischemic or nonischemic cardiomyopathy and had a positive linear correlation with C-reactive protein and a negative linear correlation with LVEF. In nonischemic cardiomyopathy patients, the CP value was an independent biomarker associated with the extent of heart failure. Further studies should be applied to confirm our data in a larger number of patients and to clarify the mechanism.

## Figures and Tables

**Table 1:** Baseline characteristics of the study population between groups.

*n* (296)	Control (94)	Ischemic cardiomyopathy (78)	Nonischemic cardiomyopathy (124)
Age (year)	43.67 ± 15.80	69.97 ± 11.05*	66.30 ± 15.23*
Male sex [*n* (%)]	43 (45.7%)	40 (51.3%)	65 (52.4%)
Smoking [*n* (%)]	19 (20.2%)	33 (42.3%)	34 (27.4%)^†^
Alcohol [*n* (%)]	10 (10.6%)	16 (20.5%)	21 (16.9%)
Hypertension [*n* (%)]	18 (19.1%)	51 (65.4%)*	65 (52.4%)*
Diabetes mellitus [*n* (%)]	0 (0%)	21 (26.9%)*	18 (14.5%)^∗†^
BMI (kg/m^2^)	22.35 ± 2.77	22.32 ± 4.52	21.09 ± 5.71
ALT (U/L)	18.82 ± 10.93	20.30 ± 11.80	31.33 ± 50.15*
AST (U/L)	21.63 ± 8.20	26.17 ± 22.98	37.19 ± 56.13*
Total cholesterol (mg/dL)	170.78 ± 37.68	158.37 ± 44.85	155.53 ± 41.18*
LDL-C (mg/dL)	100.47 ± 28.92	93.92 ± 35.86	90.84 ± 31.14*
Triglycerides (mg/dL)	114.96 ± 64.85	134.37 ± 76.33	111.74 ± 59.79^†^
Creatinine (mg/dL)	0.72 ± 0.19	0.94 ± 0.52*	1.01 ± 0.78*
Uric acid (mg/dL)	5.33 ± 1.47	6.22 ± 2.22*	6.96 ± 2.53^∗†^
CK (IU/l)	74.78 ± 32.33	100.35 ± 186.27	100.14 ± 127.47
CK-MB (IU/L)	10.60 ± 3.34	15.61 ± 27.07*	13.66 ± 6.91
TNI (ng/mL)	0.06 ± 0.21	0.66 ± 3.75	0.28 ± 1.79
CRP (mg/L)	3.85 ± 7.99	14.77 ± 24.84*	13.80 ± 27.27*
Homocysteic acid (*μ*mol/L)	12.25 ± 5.49	16.66 ± 7.15*	17.12 ± 10.22*
LVEF (%)	63.96 ± 4.85	53.29 ± 14.36*	48.58 ± 15.52^∗†^
LVIDd (cm)	4.55 ± 0.42	5.19 ± 0.95*	5.60 ± 1.11^∗†^
LA (cm)	3.09 ± 0.38	3.93 ± 0.67*	4.47 ± 1.01^∗†^
Cardiac tonic [*n* (%)]	0 (0%)	13 (16.7%)*	68 (54.8%)^∗†^
Diuretic [*n* (%)]	0 (0%)	37 (47.4%)*	100 (80.6%)^∗†^
Nitrate [*n* (%)]	0 (0%)	56 (71.8%)*	83 (66.9%)*
ACEI/ARB [*n* (%)]	9 (9.6%)	72 (92.3%)*	105 (84.7%)*
Beta blockers [*n* (%)]	9 (9.6%)	56 (71.8%)*	83 (66.9%)*
CCB [*n* (%)]	11 (11.7%)	29 (37.2%)*	30 (24.2%)^∗†^
Aspirin/clopidogrel [*n* (%)]	4 (4.3%)	74 (94.9%)*	71 (57.3%)^∗†^
Statins [*n* (%)]	2 (2.1%)	72 (92.3%)*	23 (18.5%)^∗†^

ACEI: angiotensin-converting enzyme inhibitor; ALT: alanine aminotransferase; ARB: angiotensin receptor blocker; AST: aspartate aminotransferase; BMI: body mass index; CCB: calcium channel blocker; CK: creatinine kinase; CK-MB: creatinine kinase-MB; CRP: C-reactive protein; HDL-C: high-density lipoprotein cholesterol; LA: left atrium; LDL-C: low-density lipoprotein cholesterol; LVEF: left-ventricular ejection fraction; LVIDd: left-ventricular internal diameter at end diastole; TNI: troponin I.

*P < 0.05 versus control group.

^†^ *P* < 0.05 versus ischemic cardiomyopathy group.

**Table 2:** Linear correlation between ceruloplasmin and other parameters.

	Pearson correlation	*P* value
Creatinine	−0.028	0.635
ALT	0.229	<0.001*
AST	0.286	<0.001*
LDL-c	−0.047	0.428
Total cholesterol	−0.083	0.160
Triglycerides	−0.061	0.301
Uric acid	0.249	<0.001*
CK	0.046	0.435
CKMB	0.037	0.553
Troponin I	0.006	0.926
CRP	0.449	<0.001*
Homocysteic acid	0.094	0.107
LVEF	−0.151	0.032*
LVIDd	0.213	0.003*
LA	0.215	0.003*

ALT: alanine aminotransferase; AST: aspartate aminotransferase; CK: creatinine kinase; CK-MB: creatinine kinase-MB; CRP: C-reactive protein; LA: left atrium; LDL-C: low-density lipoprotein cholesterol; LVEF: left-ventricular ejection fraction; LVIDd: left-ventricular internal diameter at end diastole.

*P < 0.05.

**Table 3:** Baseline characteristics of patients with nonischemic cardiomyopathy.

	Nonischemic cardiomyopathy (124)			
	NYHA II (21)	NYHA III (71)	NYHA IV (32)	*P* value
Age (year)	68.52 ± 16.23	63.69 ± 15.49	70.00 ± 13.32	0.110
Male sex [*n* (%)]	10 (47.6%)	42 (59.2%)	13 (40.6%)	0.195
Smoking [*n* (%)]	5 (23.8%)	24 (33.8%)	6 (18.8%)	0.258
Alcohol [*n* (%)]	4 (19.0%)	15 (21.1%)	3 (9.4%)	0.347
Hypertension [*n* (%)]	12 (57.1%)	34 (47.9%)	19 (59.4%)	0.498
Diabetes mellitus [*n* (%)]	4 (19.0%)	7 (9.9%)	7 (21.9%)	0.225
BMI (kg/m^2^)	21.56 ± 3.11	21.29 ± 5.68	20.12 ± 7.41	0.639
ALT (U/L)	17.33 ± 12.50	27.60 ± 26.02	48.69 ± 88.41	0.052
AST (U/L)	23.14 ± 6.98	31.14 ± 22.77	59.84 ± 102.72	0.024*
Total cholesterol (mg/dL)	165.81 ± 50.27	154.94 ± 39.12	151.65 ± 39.59	0.460
LDL-C (mg/dL)	94.52 ± 40.68	90.74 ± 28.34	89.23 ± 30.50	0.832
Triglycerides (mg/dL)	123.05 ± 77.44	112.51 ± 60.00	104.13 ± 44.42	0.537
Creatinine (mg/dL)	0.83 ± 0.25	0.95 ± 0.40	1.01 ± 0.47	0.292
Uric acid (mg/dL)	5.92 ± 1.82	6.96 ± 2.04	7.71 ± 3.55	0.044*
CK (IU/L)	93.70 ± 84.26	90.88 ± 135.05	124.97 ± 133.91	0.451
CK-MB (IU/L)	10.26 ± 3.72	13.05 ± 6.50	17.17 ± 7.89	0.001*
Troponin I (ng/mL)	0.02 ± 0.01	0.13 ± 0.77	0.77 ± 3.25	0.213
CRP (mg/L)	4.98 ± 4.43	9.11 ± 12.07	27.43 ± 45.96	0.002*
Homocysteic acid (*μ*mol/L)	15.11 ± 5.10	17.65 ± 12.10	17.25 ± 7.89	0.609
LVEF (%)	61.53 ± 8.54	47.14 ± 15.73	46.80 ± 31.06	0.022*
LVIDd (cm)	5.09 ± 0.93	5.68 ± 1.08	5.74 ± 1.24	0.101
LA (cm)	4.23 ± 0.52	4.47 ± 1.11	4.63 ± 1.00	0.434
Cardiac tonic [*n* (%)]	6 (28.6%)	40 (56.3%)	22 (68.8%)	0.017*
Diuretic [*n* (%)]	7 (33.3%)	64 (90.1%)	30 (93.8%)	<0.001*
Nitrate [*n* (%)]	9 (42.9%)	49 (69.0%)	25 (78.1%)	0.024*
ACEI/ARB [*n* (%)]	15 (71.4%)	64 (90.1%)	27 (84.4%)	0.099
Beta blockers [*n* (%)]	12 (57.1%)	53 (74.6%)	18 (56.2%)	0.107
CCB [*n* (%)]	7 (33.3%)	12 (16.9%)	10 (31.2%)	0.140
Aspirin/clopidogrel [*n* (%)]	12 (57.1%)	40 (56.3%)	18 (56.2%)	0.998
Statins [*n* (%)]	5 (23.8%)	14 (19.7%)	4 (12.5%)	0.542

ACEI: angiotensin-converting enzyme inhibitor; ALT: alanine aminotransferase; ARB: angiotensin receptor blocker; AST: aspartate aminotransferase; BMI: body mass index; CCB: calcium channel blocker; CK: creatinine kinase; CK-MB: creatinine kinase-MB; CRP: C-reactive protein; HDL-C: high-density lipoprotein cholesterol; LA: left atrium; LDL-C: low-density lipoprotein cholesterol; LVEF: left-ventricular ejection fraction; LVIDd: left-ventricular internal diameter at end diastole.

*P < 0.05 among NYHA II, NYHA III, and NYHA IV subgroups.

**Table 4:** Relationship between extent of heart failure and clinical and laboratory parameters in patients with nonischemic cardiomyopathy.

Variables	OR	*χ* ^2^	*P* value	95% confidence interval	
				Lower bound	Upper bound
Age	1.072	3.997	0.046*	1.001	1.148
Gender	0.293	1.426	0.232	0.039	2.198
Smoking	1.499	0.172	0.678	0.222	10.136
Alcohol	0.993	0.000	0.994	0.125	7.874
Hypertension	2.330	1.214	0.270	0.518	10.492
Diabetes mellitus	1.047	0.002	0.967	0.121	9.026
Creatinine	0.157	1.029	0.310	0.004	5.609
ALT	1.019	0.339	0.560	0.957	1.084
AST	0.972	0.700	0.403	0.910	1.039
LDL-C	1.026	0.481	0.488	0.954	1.103
Total cholesterol	0.981	0.621	0.431	0.935	1.029
Triglycerides	0.998	0.076	0.782	0.981	1.014
Uric Acid	1.141	0.530	0.467	0.800	1.629
CK	1.006	1.962	0.161	0.998	1.013
CKMB	1.069	0.937	0.333	0.934	1.223
Troponin I	1.304	1.011	0.315	0.778	2.185
CRP	1.012	0.255	0.614	0.966	1.061
Homocysteic acid	1.009	0.057	0.811	0.937	1.087
LVEF	0.981	1.479	0.224	0.952	1.012
LVIDd	0.616	0.502	0.479	0.161	2,354
LA	0.839	0.097	0.755	0.280	2.519
Ceruloplasmin	1.010	4.489	0.034*	1.001	1.019

ALT: alanine aminotransferase; AST: aspartate aminotransferase; CK: creatinine kinase; CK-MB: creatinine kinase-MB; CRP: C-reactive protein; LA: left atrium; LDL-C: low-density lipoprotein cholesterol; LVEF: left-ventricular ejection fraction; LVIDd: left-ventricular internal diameter at end diastole. *P < 0.05.
